# Prosociality and health: Identification with all humanity is a replicable predictor of prosocial motivation for health behaviors

**DOI:** 10.3389/fpsyg.2022.1052713

**Published:** 2023-01-12

**Authors:** Rodolfo Cortes Barragan, Andrew N. Meltzoff

**Affiliations:** ^1^Institute for Learning and Brain Sciences, University of Washington, Seattle, WA, United States; ^2^Department of Psychology, University of Washington, Seattle, WA, United States

**Keywords:** prosociality, identification with all humanity, COVID-19, mask-wearing, physical distancing, social interaction, social cognition, ingroups

## Abstract

The acute phase of the COVID-19 pandemic may have passed, but the pandemic remains a major worldwide health concern that demands continued vigilance. Are there individual differences that predict the motivation to continue to wear masks and to create physical distance in public? Previous research conducted early in the pandemic had suggested that a particular social identity—*identification with all humanity*—is one underlying factor that contributes to people’s cooperation with health behavior guidelines. This highlights that the pandemic is not only an issue to be tackled with the tools of immunology and epidemiology. It also requires the tools from psychology—to measure the representations people have about themselves and others and how these representations drive values and decisions related to health. Here we report work on U.S. respondents that examined whether individuals’ level of identification with all humanity predicts their prosocial health behaviors aimed at mitigating the spread of COVID-19. In 3 convergent studies (total *N* = 1,580), we find that identification with all humanity predicted the prosocial motivation to wear masks and to engage in physical distancing when in public without a mask. The results were obtained while controlling for a host of covariates, including demographics, educational attainment, and Big Five personality dimensions. We find that some people have a marked drive to care for the health of strangers, which is significantly linked to their concern for *all humanity* rather than being restricted to their care for their community or country. Discussion focuses on this social identification with humanity and its enduring, replicable role in predicting the motivation to engage in prosocial health behaviors. We note key implications for theories in social and developmental psychology as well as for research that may lead to practical applications for lessening the human toll of the current and future pandemics.

## 1. Introduction

The COVID-19 pandemic has been one of the most pressing challenges to global society in the early 21^st^ century—and there are considerable barriers to its mitigation. On September, 14^th^, 2022, *The Lancet* COVID-19 Commission concluded that the unprecedented human and economic costs of the pandemic could be traced in large part to issues of prosociality (Sachs et al., 2022; The Lancet, 2022). Often defined as actions that benefit others, prosociality is a key concept in the biological and social sciences (Keltner et al., 2014). In the context of addressing and ending the pandemic, the Commission argued that prosociality would involve a reorientation of priorities from meeting the needs of individuals toward meeting the needs of society as a whole. As noted by Sachs et al. (2022), societies are organized into levels—ranging from the individual, to national, to global levels—and prosociality to combat COVID-19 can occur both within and between these levels. This view holds that governments can empower individuals to engage in prosocial health behaviors aimed at mitigating COVID-19, and individuals can take responsibility for carrying out such behavior, inasmuch as they feel it is the right thing to do. An important question then becomes, *who* is likely to proactively enact such health behaviors, even in the absence or reduction of governmental guidelines and legal enforcements?

Peoples’ social groups and identities are important for motivating their behavior (Walton and Cohen, 2011). Individuals generally tend toward identifying and engaging prosocially with their own immediate social group (Tajfel and Turner, 1979), often called ingroup favoritism. However, there has also been a historical process that has led people toward identifying at broader levels. As discussed by McFarland (2011), especially since the beginning of the 20^th^ century, there has been a trend whereby people are becoming increasingly identified with “all humanity.” This concept of stretching our ingroup to a wider circle of humans—perhaps to members of the human species in general—has implications for both our explicit reflections about moral obligations and also for our quick, gut-level feelings of connectivity to our fellow travelers. This psychological process may then influence our everyday public health behaviors.

Data collected by Barragan et al. (2021) in the first months of the pandemic showed that individuals around the world were generally reliant on government mandates guided by the World Health Organization (WHO). In the initial months of the pandemic, U.S. public health messaging was aimed toward recommending/requiring people to stay home to “flatten the curve”—a behavior aimed especially toward helping health workers manage COVID-19 hospitalizations (Block et al., 2020). But as the pandemic unfolded, public support for these health behaviors began to fragment, with some people continuing to comply with the recommendations/requirements (e.g., staying home, mask wearing, social distancing) and others rejecting the continuation of these behaviors. Some regarded these health behaviors as a violation of their individual liberties (Studdert and Hall, 2020). Building on the proposals by McFarland (2011), here we suggest that a key individual predictor of *who* will continue to engage in health behaviors aimed at mitigating the spread of COVID-19—even as specific governmentally mandated requirements are relaxed—involves a feeling of *identification with all humanity*.

The felt obligations or connectivity to fellow humans, even strangers, was investigated in the classic studies of rescuers in Nazi-occupied Europe—people who voluntarily placed themselves at grave personal risk in order to help strangers survive (Oliner and Oliner, 1988; Monroe, 1996). Rescuers explained the motivation behind their own actions as a self-imposed commitment to extend prosociality toward all humans. One rescuer articulated this drive as follows: “I sensed I had in front of me human beings being hunted down like wild animals. This aroused a feeling of brotherhood with the desire to help” (Oliner, 2002, p. 125). Such prosociality toward strangers (“heroic altruism”) is an important topic in social psychology (Batson, 2011), and it is illuminating to examine this motive and its expression in the context of the prosocial behaviors needed to mitigate the current pandemic. In the COVID-19 context, we acknowledge that the threat is not to a specific group of humans based on religion, ethnicity, or other minority status as it was in Nazi-occupied Europe. Rather, the threat derives from a virus that “hunts” all humans and kills, or saps the health of, those it strikes. In such a context, contemporary individuals have an opportunity to engage in “a feeling of brotherhood” by participating in health behaviors that can help to curtail the spread of the coronavirus to others, including strangers.

### 1.1. Identification with all humanity and prosocial health behaviors during COVID-19

Using the identification with all humanity scale developed by McFarland et al. (2012), empirical studies conducted prior to the pandemic, and in other more typical contexts, show that this psychological scale uniquely predicts a range of prosocial actions in a variety of situations (e.g., McFarland et al., 2013; Reese et al., 2015; Hamer et al., 2019). In the first study on identification with all humanity conducted during the pandemic, Barragan et al. (2021) collected data on respondents from 87 countries during the initial outbreak of the crisis (April–June 2020). The results demonstrated that identification with all humanity was the strongest predictor of (i) adopting the health behaviors recommended by WHO and (ii) showing prosociality toward others on a variety of experimentally presented scenarios, e.g., donating masks to a hospital in need, helping people who exhibited COVID-19 symptoms. Building on this work, Sparkman (2022) used a sample of U.S. respondents and showed that in August 2020, identification with all humanity predicted cooperation with recommended health behaviors, and the effect was robust to many of the same (and more) covariates as used in Barragan et al. (2021). The effect of identification with all humanity on cooperation with recommended health behaviors was maintained into the first “winter crest” of COVID-19 infection, as demonstrated in Marchlewska et al.’s (2022) report that in December of 2020, identification with all humanity significantly predicted cooperation with newly emerging governmental recommendations for combating the pandemic, i.e., willingness to vaccinate.

As the pandemic extended into its second year, national and local government health agencies relaxed or dropped their requests for local governments and individuals to cooperate with previously recommended health behaviors, notably masking and social distancing (Centers for Disease Control and Prevention, 2022). In the U.S., this meant that the continued enactment of these behaviors became less a decision to cooperate with guidelines and laws, and more of a personal choice. Despite the changes to messaging by the government, many individuals may feel motivated to continue to engage in the previously recommended health behaviors to prevent the spread of the disease—often for the sake of strangers, and at some cost to the self. We hypothesized that, in the context of lapsed health guides of early autumn of 2022, the identification with humanity might account for peoples’ continued enactment of health behavior that is aimed at benefitting others even while controlling for other likely covariates. That is, whereas in previous phases of the pandemic, enactment of health behaviors could be interpreted as conforming behavior to recommended and legally-mandated actions, peoples’ continuance of these behaviors (in spite of the expiration of mandates and the relaxation of guidelines) may have a strong *prosocial* component: wearing a mask and engaging in social distancing for the sake of protecting strangers.

### 1.2. Current studies

We collected the data from U.S. respondents during the interval between September 8^th^-September 13^th^ 2022. Study 1 was conducted on Amazon Mechanical Turk, a method often used in psychology research studies (Paolacci and Chandler, 2014). Study 2 was also carried out on Turk, as an exact replication (Brandt et al., 2014). Study 3 was carried out on the Prolific Academic platform, with the purpose of testing generalizability, inasmuch as the demographics of participants on Turk and Prolific are known to be different (see below). According to scientific studies of the two recruitment pools, respondents on Prolific are more racially diverse than respondents on Turk (Peer et al., 2017). Moreover, respondents on Prolific are less experienced with taking psychology surveys than participants on Turk (Palan and Schitter, 2018). Finally, Prolific uses an algorithm to obtain a “representative sample” of respondents in terms of age, sex, and ethnicity (Prolific Team, 2022), which we used. We thus reasoned that an initial attempt at generalizability could be achieved by conducting the study not only on Turk but also on Prolific. As in related research (Barragan et al., 2021; Marchlewska et al., 2022; Sparkman, 2022), all analyses were conducted using regression.

## 2. Study 1

### 2.1. Sample size and power analyses

Using G*Power (Faul et al., 2007) with pilot data, we found that a sample size of *N* = 176 respondents would afford 80% power for detecting a small- to medium-sized effect of identification with all humanity on prosocial outcome variables, assuming an alpha of 0.05 (2-tailed tests) and a multiple regression analysis plan. In order to align with calls within social and personality psychology toward larger samples than required by power analyses (Fraley and Vazire, 2014), we predetermined to request exactly 575 respondents from the Amazon Turk platform. This prespecified number was expected to yield an analytic sample size of approximately 500, which would place our study among the top 5–10% of sample sizes in the field (Sassenberg and Ditrich, 2019). We also requested exactly *n* = 575 for each of the replications (Study 2 and 3), so that the stopping rule was the same for all studies.

### 2.2. Participants, methods, and procedure

Out of an original 575 respondents, the analytic sample was *n* = 553 participants. Excluded respondents were those who did not provide a valid Amazon Turk ID, had participated in the pilot study, or had taken the survey more than one time (only their first submission was retained). The demographics of the analytic sample are shown on Table 1. The survey was constructed using Qualtrics.

**Table 1:** Descriptive statistics for predictor and outcome variables in Study 1, 2, and 3.

	Study 1 (*N* = 553)		Study 2 (*N* = 485)		Study 3 (*N* = 542)	
Variables	*M* or %	*SD*	*M* or %	*SD*	*M* or %	*SD*
Predictors						
Age (years)	36.06	10.77	36.83	10.46	45.98	16.57
Gender (% female)	42.13		40.62		49.45	
Race (% White)	86.26		77.94		73.43	
Education	7.31	1.20	7.29	1.15	6.85	1.34
Income	3.22	0.81	3.19	0.82	2.71	0.92
Risk (% high risk)	54.61		48.04		19.93	
Conservativism	3.97	2.12	3.98	2.12	3.26	1.71
Extraversion	3.93	1.17	3.96	1.10	3.24	1.68
Agreeableness	4.34	1.05	4.53	1.06	5.33	1.20
Conscientiousness	4.52	1.13	4.64	1.17	5.45	1.29
Stability	4.42	1.11	4.56	1.22	4.81	1.59
Openness	4.30	1.06	4.36	1.07	4.99	1.27
Community	3.87	0.75	3.88	0.71	3.64	0.86
Nation	3.94	0.75	3.90	0.77	3.52	0.85
Humanity	3.96	0.80	3.93	0.81	3.59	0.87
Outcome						
Prosocial health behaviors	4.07	0.87	4.09	0.83	3.56	1.34

### 2.3. Measures

With the exception of the block of questions about demographics, which always appeared first, the remaining five blocks of questions were administered in a random order.

**Demographics**. We measured five standard demographic variables: Age, gender, race/ethnicity, educational attainment, and household income. For age, respondents clicked on the number representing their age in years. For gender, the choices were female, male, non-binary, prefer to self-describe (text response), and prefer not to say; responses were effect coded as female (+1) or non-female (−1; consistent with coding used in Barragan et al., 2021; see also Galasso et al., 2020). Race/ethnicity were assessed by asking participants to select all options that may apply: American Indian or Alaskan Native, Asian, Black or African-American, Hispanic or Latin, Native Hawaiian or Other Pacific Islander, White, and Other. Responses were coded as White only (+1) or non-White (−1; Williams et al., 2022, suggest White/non-White differences in COVID-19 concerns). Educational attainment was measured on a 9-point scale: No formal education, incomplete primary school, completed primary school, incomplete secondary school, completed secondary school, incomplete college (no degree), completed college (obtained degree), incomplete graduate/professional school (no degree), or completed graduate/professional school (obtained degree). Household income, following the General Social Survey (Marsden et al., 2020), was measured on a 5-point Likert-type scale by asking participants to rate their household income in comparison to U.S. households in general (1 = “Far below average” to 5 “Far above average”).

**Big Five Personality Dimensions**. The 10-Item Personality Inventory (TIPI) is a short inventory designed for research in which personality is not the central topic of interest (Gosling et al., 2003). It assesses the Big Five personality dimensions (agreeableness, conscientiousness, emotional stability, extraversion, and openness to experience). Participants were asked to consider the extent to which short descriptions of personality dimensions apply to themselves (e.g., “reserved, quiet,” “disorganized, careless”), and responses are measured using a 7-point Likert-type scale (1 = “Strongly disagree” to 7 “Agree strongly”).

**Political Ideology**. Participants were asked to place themselves on a 7-point Likert-type scale (1 = “Very liberal” to 7 “Very conservative”).

**COVID-19 High Risk**. Using the same measure as in prior research (Barragan et al., 2021), we asked participants “Do you consider yourself to be at high risk for severe illness if infected with COVID-19?” Participants selected yes (coded as +1), no, or not sure (coded as −1).

**Identification with Community, Nation, and All Humanity**. Identification with community, nation, and humanity are related psychological tendencies that can be assessed together (McFarland et al., 2012). Prior work established that four items of the original scale can be considered a subfactor that is especially relevant for predicting prosociality (Reese et al., 2015; Sparkman and Hamer, 2020; Hamer et al., 2021; Sparkman, 2022). The four items isolated by that work, which are each asked separately in regard to community, nation, or humanity are: (i) “How much do you want to be a responsible citizen of your community (identification with community)/your country (identification with nation)/the world (identification with all humanity)?” (ii) “How much do you believe in being loyal to my community (identification with community)/ my country (identification with nation)/all humanity (identification with all humanity)?” (iii) “How much would you say you care (feel upset, want to help) when bad things happen to people in my community (identification with community)/my country (identification with nation)/all over the world? (identification with all humanity)” (iv) “When they are in need, how much do you want to help people in my community (identification with community)/people in my country (identification with nation)/people all over the world (identification with all humanity)?” As in prior work (McFarland et al., 2012; Barragan et al., 2021), we had respondents rate the extent to which each of these items applied to themselves using a 5-point Likert-type scale (1 = “Not at all” to 5 = “Very much”). For each respondent’s ratings, average scores were calculated for the “identification with community” variable (4 community items), the “identification with nation” variable (4 country items), and the “identification with all humanity” variable (4 world items). As in McFarland et al.’s (2012) original work in scale development, good internal consistency was achieved for each scale: The identification with community scale (Cronbach’s α = 0.86), the identification with nation scale (Cronbach’s α = 0.81), and the identification with all humanity scale (Cronbach’s α = 0.84). The score analyzed was the mean of the four items for each of the three scales (identification with community, identification with nation, identification with all humanity). Our chief hypothesis pertained to the identification with all humanity scale, however prior work has also shown interesting effects for identifications with community and nation and so we thought it was useful to include all three constructs.

**Prosocial Motivation for Health Behaviors**. We constructed a measure tapping prosocial health behaviors. One item was *prosocial motivation to wear masks* (“How important is it to wear a mask to protect strangers in public?”) measured on a 5-point Likert-type scale (1 = “Not at all important” to 5 = “Extremely important”). A second item was *prosocial motivation for physical distancing to avoid spreading COVID-19* (“If you find yourself in public without a mask, how motivated are you to increase your physical distance from other people to avoid spreading COVID-19?”) measured on a 5-point Likert-type scale (1 = “Not at all” to 5 = “Extremely”). The presentation order of the two items was randomized. The items had good internal consistency (Cronbach’s α = 0.74) and were averaged together into a single index of prosocial motivation for health behaviors.

### 2.4. Results

As hypothesized, identification with all humanity significantly predicted prosocial motivation for health behaviors (*b* = 0.29, *b* s.e. = 0.04, *t* = 6.92, *p* = 1.3^−11^). The identification with community and high risk variables were also significant predictors and no other variable was a significant predictor (see Table 2).

**Table 2:** Multiple regression analysis using identification with all humanity predicting prosocial motivation for health behaviors in Study 1, 2, and 3.

	Study 1 (*N* = 553)			Study 2 (*N* = 485)			Study 3 (*N* = 542)		
Predictors	*b*	*b SE*	*p*	*b*	*b SE*	*p*	*b*	*b SE*	*p*
Age	−0.03	0.03	0.379	0.03	0.03	0.308	0.09	0.06	0.137
Female	0.00	0.03	0.946	0.07	0.03	0.021	0.10	0.05	0.039
White	0.03	0.05	0.493	−0.07	0.04	0.069	−0.19	0.06	<0.001
Education	0.03	0.03	0.422	−0.06	0.03	0.047	0.02	0.05	0.687
Income	0.01	0.03	0.816	−0.02	0.03	0.456	−0.03	0.05	0.508
Risk	0.12	0.03	<0.001	0.08	0.03	0.015	0.30	0.06	<0.001
Conservativism	−0.04	0.03	0.214	−0.01	0.03	0.647	−0.48	0.06	<0.001
Extraversion	0.02	0.03	0.608	−0.07	0.03	0.043	−0.10	0.06	0.086
Agreeableness	0.00	0.04	0.975	0.02	0.04	0.561	0.02	0.06	0.722
Conscientiousness	−0.05	0.05	0.288	−0.06	0.04	0.154	0.06	0.06	0.316
Stability	−0.02	0.04	0.580	−0.05	0.04	0.202	−0.14	0.06	0.027
Openness	0.05	0.04	0.216	0.13	0.04	<0.001	0.09	0.06	0.101
Community	0.18	0.04	<0.001	0.19	0.05	<0.001	0.12	0.09	0.174
Nation	0.05	0.04	0.257	0.13	0.04	0.002	0.05	0.09	0.566
Humanity	0.29	0.04	<0.001	0.23	0.04	<0.001	0.20	0.07	0.008
Model summary	*F*(15, 537) = 19.61 *R*^2^ = 0.35, *p* < 0.001			*F*(15, 469) = 20.14 *R*^2^ = 0.39, *p* < 0.001			*F*(15, 526) = 18.53 *R*^2^ = 0.35, *p* < 0.001		

In Study 1, all VIFs < 3, tolerance > 0.40. In Study 2, all VIFs < 3, tolerance > 0.40. In Study 3, all VIFs < 4, tolerance > 0.25.

## 3. Study 2

Study 2 was an attempt to conduct an exact replication of Study 1, and was run after data collection for Study 1 had been completed. See Brandt et al. (2014) for motivation(s) for replications in psychological sciences.

### 3.1. Participants and procedure

As in Study 1, we requested 575 respondents. The resulting analytic sample was composed of *n* = 485 participants. Respondents who were not included in the analytic sample were those who did not provide a valid Amazon Turk ID, were in the pilot study, or were identified as a repeat respondent (only their first submission was retained). The demographics are shown on Table 1.

### 3.2. Results

As hypothesized, identification with all humanity significantly predicted prosocial motivation for health behaviors (*b* = 0.23, *b* s.e. = 0.04, *t* = 5.33, *p* = 1.5^−7^). Identification with community and nation were also significant, as were being female, being less educated, high risk, less extraverted, more open to experience (see Table 2).

## 4. Study 3

Study 3 conducted an extension of Study 1 and 2, by using the same survey questions with the somewhat different population afforded by the Prolific platform, which has previously been used with research on identification with all humanity early in the pandemic (Sparkman, 2022). This platform allows the researcher to elect an option for a “representative” U.S. sample, which we selected. Of course, that option was not available in Study 1 or 2, conducted on Turk.

### 4.1. Participants and procedure

As in Study 1 and 2, we requested 575 respondents. The analytic sample was composed of 542 participants. Respondents who were not included in the analytic sample were those who did not provide a valid Prolific ID, were identified as a pilot respondent, or were identified as a repeat respondent (only their first submission was retained). The demographics are shown on Table 1.

### 4.2. Results

As hypothesized, identification with all humanity predicted prosocial motivation for health behaviors (*b* = 0.20, *b* s.e. = 0.07, *t* = 2.65, *p* = 0.0083). Being female, non-White, high risk, low in emotional stability, and not conservative were also significant predictors of prosocial motivation for health behaviors (Table 2).

## 5. Cross-study effect sizes

**Figure 1:**
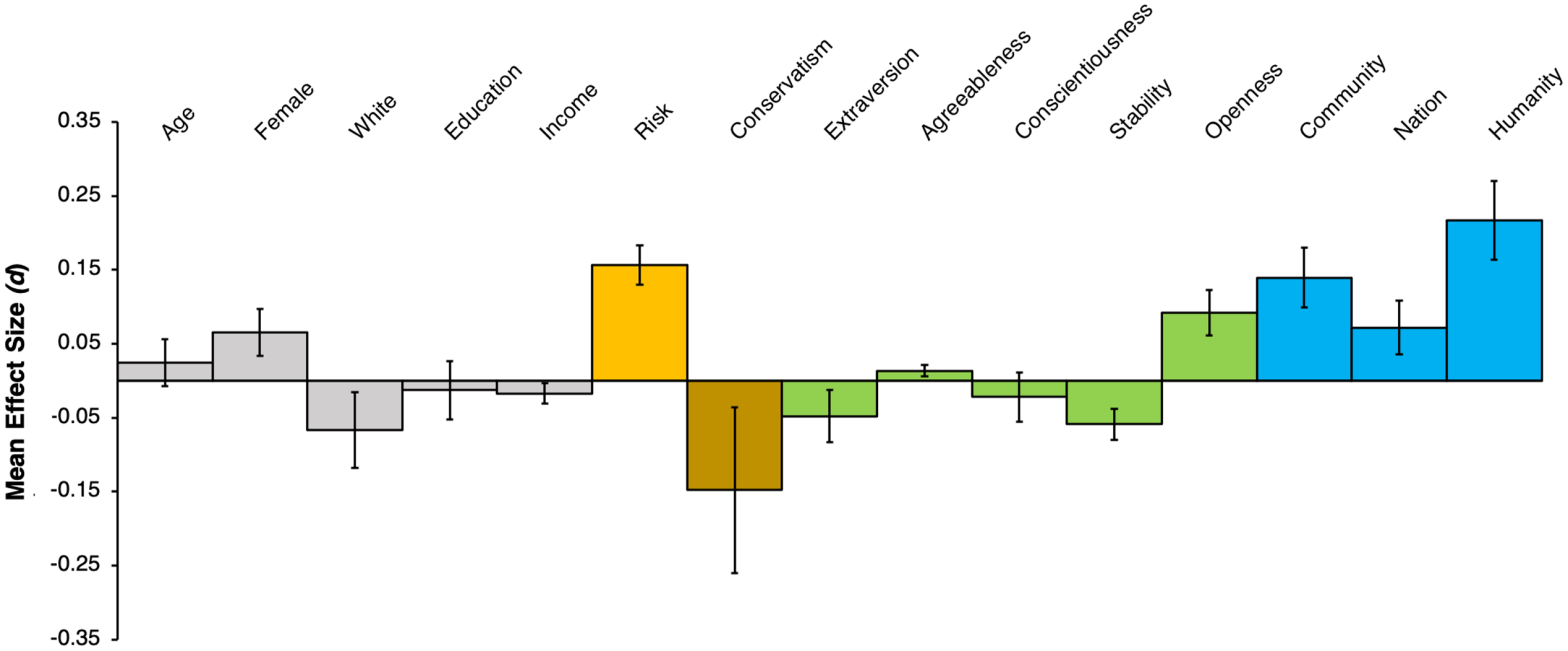
Mean effect sizes (estimated Cohen’s *d*) of predictor variables for prosocial health behaviors across three studies. The predictors are grouped into five classes (indicated by color): demographics (grey), high risk (orange), conservativism (brown), Big 5 personality dimensions (green), identification with community, nation, and all humanity (blue). See main text for definitions of each variable. Positive values indicate that the predictor is associated with a higher score for the prosocial health behavior outcome measure. Error bars represent the standard error of the mean effect size.

Following prior research (Barragan et al., 2021), we computed the mean effect sizes (Cohen’s *d*) across the three studies. To accomplish this, we computed *d* for each predictor in each study by dividing the predictor coefficient by the product of the coefficient standard error and the square root of the sample size. Then, across the studies, we computed the mean effect size and standard error of the effect size for each predictor. These mean effect sizes are displayed in Figure 1. The patterns showed that identification with all humanity had the largest positive effect size of all of the 15 predictors. COVID-19 high risk was the second largest positive effect size for each outcome, while conservatism was the largest negative effect size.

## 6. General discussion

Consistent with the perspective that identification with all humanity is a unique social identity predictor of prosociality (McFarland et al., 2012; Hamer et al., 2019; Barragan et al., 2021; Sparkman, 2022)—one that is perhaps more consequential than other “less abstract” social identities, i.e., community, nation—we found that identification with all humanity significantly predicted prosocial motivation for health behaviors during COVID-19. Moreover, this was the case while controlling for demographics (age, gender, etc.), personality, political ideology, and other covariates. Of special note is that this paper also incorporated a direct replication and a (modest) generalization study. Consistent with calls within social psychology for faithful replications (Brandt et al., 2014), Study 2 was an exact replication of Study 1 using the same survey questions and platform (Amazon Turk), and Study 3 was an initial attempt to assess whether the effects would generalize to the Prolific platform, which is known to be more racially diverse. These findings thus contribute to the movement to conduct replication/generalizability studies in psychological research (Open Science Collaboration, 2015), and show that identification with all humanity is a psychological tendency that has a robust connection with the motivation to engage prosocially with strangers—whereas none of the other variables was as consistently predictive.

### 6.1. Examining psychological contributors to prosocial health behaviors

It is known that, over the long term, isolated actions can accumulate into consistent patterns of behavior (Cohen and Sherman, 2014; Funder and Ozer, 2019). In the case of the current research, a person highly identified with all humanity may engage in relatively more prosocial health behaviors, e.g., creating physical distance when in public and without a mask, than a person with lower identification. When these actions are repeatedly done by independent individuals, such prosocial behaviors benefit public health and society as a whole. Indeed, these patterns contribute to the emerging idea that there may exist an “ideology” that emphasizes prosociality toward collectives (Nezlek, 2022). In this case, the collective of “all humanity.”

In addition to identification with all humanity, we found that high perceived risk from COVID-19 complications also predicted prosocial motivation for health behaviors (Table 2), a pattern we also found in the first months of the pandemic (Barragan et al., 2021). Why would this be the case? One possibility is that many participants may seek only to protect themselves, perhaps due to fear of COVID-19 that induces a perception of risk to themselves (Feng et al., 2022). Another related possibility is that perceived high risk for the *self* may enhance the individual’s proclivity for understanding the risk for *others*. Such a psychological process is consistent with the idea that social cognition involves seeing one’s own self in others—the view that others are, or should be, “like-me,” (e.g., Meltzoff, 2007, 2013). This “like-me” representation of others is first manifest prelinguistically in infant behavior and also is deeply-rooted human brain functioning (Meltzoff and Marshall, 2020). Building on this idea, we speculate that people who reflect on their own susceptibility for COVID-19 and its possible lifelong complications (Crook et al., 2021) may have experiences, thoughts, and feelings that they generalize or extend to others using an implicit practical syllogism: “me = vulnerable,” “others are like me,” thus “others = vulnerable warranting protection.” In this manner, the self may seek to maximize the health of humanity inclusive of itself, and therefore engaging in prosocial health behaviors creates an ultimate “positive externality” that benefits the whole of society. This understanding of others in terms of the self may thus support and motivate behavior that is aimed at benefiting others. These issues merit future empirical study.

Some of our results also suggest a role for political conservatism in predicting lower prosocial health behaviors, but this was significant only in Study 3 (Table 2), and therefore we do not wish to draw firm inferences about this pending further research. We acknowledge that in the U.S. context of our samples, the pandemic has been highly politicized (Gollwitzer et al., 2020) with those who identify as conservatives generally more opposed to health mandates than those identifying as liberals (DeMora et al., 2021; Baxter-King et al., 2022). This is probably not a cultural universal, given that conservative officials in some countries, e.g., Britain, played a role in enhancing their conservative constituents’ compliance with COVID-19 health recommendations (Taraktaş et al., 2022), which suggests that an association between identification with all humanity and political ideology may be sensitive to cultural context and/or influenced by other as yet unidentified factors. Nonetheless, a (potential) association may be worth pursuing (cf. Sparkman, 2022), inasmuch as it is known that conservatives and liberals often operate with different sets of values (Graham et al., 2009), and identification with all humanity is negatively correlated with at least some right-wing ideologies (Hamer et al., 2019; McFarland et al., 2019).

We come now to a question about the specificity of the reported links between all humanity and prosocial health behaviors. There were also significant effects of identification with community on prosocial health behaviors, but only in Study 1 and 2, and significant effects of identification with nation on prosocial health behaviors, but only in Study 2 (Table 2). These patterns are similar to previous patterns of data in the pandemic (Barragan et al., 2021), suggesting that although identification with one’s own community (Sparkman, 2022) and nation (Bonetto et al., 2021; Van Bavel et al., 2022) can predict prosocial health behaviors, identification with all humanity seems to be a more consistent predictor. Examining these identifications in tandem with other techniques for measuring identifications with broad social groups (Hamer et al., 2021; Carmona et al., 2022), could prove fruitful. We also note the importance of cultural context: For example, some nations have had strikingly robust governmental responses to the crisis (e.g., China, Israel, New Zealand). It may be the case that, if a nation takes such approaches and a person identifies highly with the nation, their identification with the nation may predict their prosocial health behaviors to a greater extent than might be the case in countries that do not adopt such a robust stance on the pandemic. More generally, cross-cultural work has the potential to greatly expand our theoretical perspectives. Research involving identification with all humanity is increasingly being conducted in multiple cultures (e.g., Deng, 2021; Hamer et al., 2021; Feng et al., 2022; Marchlewska et al., 2022), and it would be fruitful to examine the generalizability of the effects reported here outside the U.S. context.

An additional pattern is that we did not generally find that Big Five personality dimensions predicted prosocial COVID-19 health behaviors; the patterns were mostly non-significant, and also inconsistent (Table 2). At the same time, we note that research using more comprehensive Big Five questionnaires than used in the current study (e.g., the 60-item version, Soto and John, 2017) reported significant correlations between some of the Big Five dimensions and prosocial health behaviors during COVID-19 (Bogg and Milad, 2020; Zettler et al., 2022). This raises at least three issues. First, Big Five personality dimensions may be detectable as significant predictors of prosocial health behaviors but shortened assessments of the Big 5 may not be sufficiently sensitive. Second, it is possible that Big 5 may not be a significant predictor of prosocial health behaviors during COVID-19 when measures of social identifications and large number of covariates are taken into account in the model (as in our study). Third, other research examining the effect of personality on responding to the pandemic has suggested that different personality factors may be more or less predictive at different phases of the pandemic (Wright and Fancourt, 2021; Daniel et al., 2022). These complexities merit further testing to more closely examine how personality predicts prosocial health behaviors, under what circumstances, and when controlling for which other covariates.

### 6.2. Limitations and future directions

Although these studies have notable strengths, we acknowledge that there are a number of limitations. First, the study populations were obtained through Amazon Turk and Prolific, and the results may only be applicable to people who choose to take online surveys for the compensation offered on these platforms. As shown in Table 1, the Amazon Turk samples (Study 1 and 2) tended to have fewer women and more Whites than the Prolific sample (Study 3). Both platforms had fewer Hispanic/Latinx respondents and higher educational attainment than in the general U.S. population. For this reason, it would be judicious for future research to recruit samples that more closely mirror the general population in addition to using additional surveying methodologies, e.g., large-scale random probability sampling. Yet, despite the differences between the Amazon Turk and the Prolific samples, what is notable is that with both platforms, identification with all humanity is a significant psychological predictor of prosocial health behaviors—a finding that fits well with cross-cultural work examining constructs related to identification with all humanity (Chen et al., 2022).

Second, we did not examine the full scale of identification with all humanity. Although initially proposed as a unidimensional construct (McFarland et al., 2012), the identification with all humanity scale is increasingly considered to be comprised of two subfactors (Reese et al., 2015; Sparkman and Hamer, 2020; Hamer et al., 2021; Sparkman, 2022). In the current research, we examined the four items sometimes described as subfactor “global self-investment” or “concern for all humanity” rather than the “global self-definition” or “bond with all humanity” subfactor. As such, the current research does not address the full construct originally discussed by McFarland et al. (2012), and it is important to consider that the bond with all humanity subfactor may not consistently predict multiple dimensions of prosociality during COVID-19 (Sparkman, 2022). Rather, it is the four items forming a concern with all humanity subscale of identification with all humanity that may most consistently predict prosocial health behaviors during the pandemic.

Third, although the effect sizes in the current work could be described as “small” (Cohen, 1988), more recent meta-analyses of the literature on individual differences suggest that these effect sizes may more appropriately be classified as “medium”—typical effect sizes for individual differences research that predicts meaningful health and lifetime achievement outcomes (Gignac and Szodorai, 2016). Additionally, whether the effect is classified as “small” or “medium,” what is key is that identification with all humanity is the most robust/consequential predictor in these studies of the motivation to protect the health of strangers, suggesting the potential practical importance of the effect (see Conclusion).

Fourth, our outcome measure was a composite of two items (about masks and physical distancing) that were designed to tap salient contemporary issues and to be suitable for rapid online testing. We acknowledge that a larger and more nuanced battery of outcome measures would have been desirable to allow us, for example, to more clearly differentiate the degree to which respondents were concerned about protecting self versus protecting others. Relatedly, some of our predictor variables were single-item measures, e.g., the conservativism and the high risk variables, and it would be useful to test whether multi-item assessments of these constructs yield the same patterns. Indeed, research shows that simple, single-items scales of political ideology are problematic (Bauer et al., 2017; Wojcik et al., 2021), and it is known that political ideology has multiple dimensions, including sociocultural and economic dimensions (Johnston and Ollerenshaw, 2020). Future research involving identification with all humanity and prosocial health behaviors should take this multidimensionality into account.

Fifth, this work is purely correlational in nature. Although the studies show that identification with all humanity consistently predicts self-reported motivation for prosocial health behaviors, they do not demonstrate that identification with all humanity induces prosocial health behaviors. This begs the question: Is there a possibility that an intervention that promotes identification with all humanity will cause great prosocial health behaviors directed towards the welfare of others? Some studies suggest that identification with all humanity can be experimentally manipulated (Reese et al., 2015), but this has not always replicated (Reysen et al., 2021; Sparkman et al., 2022). Nevertheless, it is known that some social identities are modifiable through experimental treatments (Walton and Cohen, 2011; Belmi et al., 2015; Brady et al., 2020) and further experimental research would be desirable before concluding that identification with all humanity can or cannot have a causal impact on important health outcomes, such as the prosocial health behaviors measured here.

### 6.3. What are the developmental roots of identifying with all humanity?

We are particularly interested in extending this research in a new direction, towards the *child developmental origins* of identification with all humanity. This identification seems to be clearly in place by late adolescence (Albarello et al., 2021). Maslow (1970) considered adults’ feelings of affection for a wide circle of others, including strangers, as an aspect of reaching high levels of psychological well-being that might be influenced by childhood experiences. However, we know of no research directly examining the possibility that parents’ level of identification with all humanity may have a measurable effect on children’s developing prosociality (cf., Hagel et al.’s, 2022 work on people’s memories of their parents). Consistent with proposals by McFarland et al. (2013), such an intergenerational process could occur, inasmuch as it has been shown that children’s’ expression of prosociality toward others is impacted by their caregivers (e.g., Bowlby, 1969; Barragan and Meltzoff, 2021), and in the pandemic, children may be receiving both implicit and explicit messages from parents related to showing care for others by engaging in prosocial health behaviors (e.g., mask wearing). At a more abstract level, some parents engage in behaviors that promote children viewing other people, even strangers, as “like me” (Meltzoff, 2013), and this may support and enhance a basic proclivity that is present in primitive form during infancy prior to formal verbal discussions with the child (Meltzoff and Moore, 1995; Barragan and Meltzoff, 2021). Future research is needed using direct assessments of parents’ values, their verbal explanations to their children, and the children’s own behavior to probe the potential intergenerational transmission of identification with all humanity and prosociality more generally.

## 7. Conclusion

Psychology plays a considerable role in human health (Taylor et al., 1997; Fredrickson, 2001), and the present research shows peoples’ identification with all humanity is key to predicting their willingness to contribute to the health of *others*. That is, while much of social and personality psychology examines the nature and course of prosocial interactions within families, cultures, and societies (Keltner et al., 2014), the work on identification with all humanity is consistently suggesting that there is a portion of the population, across multiple cultures, that strives for showing concern not only for their kin, community, or country, but for all humans (McFarland and Hornsby, 2015; McFarland et al., 2019; Sparkman and Hamer, 2020; Barragan et al., 2021; Deng, 2021; Hamer et al., 2021; Wang et al., 2021; Feng et al., 2022; Zagefka, 2022).

We suggest that, alongside being a social identity (McFarland et al., 2012; Sparkman et al., 2022), this identification is a *generative belief*—a mental representation of accrued social-cultural-historical experience. Such broad beliefs (“mindsets”) can engender or become stable patterns of behavior, i.e., “dispositions” or “personality,” but they may also remain modifiable through environmental input (Cohen, 2003; Dweck, 2008). It is possible that future interventions may succeed in promoting both identification with all humanity as well as its (potential) prosocial behavioral sequelae. That is, by studying, understanding, and promoting identification with all humanity, societies may be able to strengthen their response to pan-human crises, including socioeconomic upheavals, climate catastrophes, and inter-nation conflicts.

## Data availability statement

The raw data supporting the conclusions of this article will be made available by the authors, without undue reservation.

## Ethics statement

The studies involving human participants were reviewed and approved by University of Washington Human Subjects Division (HSD). Written informed consent for participation was not required for this study in accordance with the national legislation and the institutional requirements.

## Author contributions

RCB and ANM conceptualized and developed the design of the studies. RCB conducted data collection and analyses. All authors contributed to the article and approved the submitted version.

## Funding

This research was supported by Templeton World Charity Foundation (Grant TWCF0520) and the Bezos Family Foundation.

## Conflict of interest

The authors declare that the research was conducted in the absence of any commercial or financial relationships that could be construed as a potential conflict of interest.

## Publisher’s note

All claims expressed in this article are solely those of the authors and do not necessarily represent those of their affiliated organizations, or those of the publisher, the editors and the reviewers. Any product that may be evaluated in this article, or claim that may be made by its manufacturer, is not guaranteed or endorsed by the publisher.
